# Field-Applicable Loop-Mediated Isothermal Amplification for the Detection of Seven Common Human Papillomavirus Subtypes

**DOI:** 10.3390/tropicalmed9100240

**Published:** 2024-10-12

**Authors:** Hongyi Li, He Tan, Xiaona Lv, Zhiqiang Han, Yuxin Wang, Shijue Gao, Ruiqin Zhang, Xinxin Shen, Xuejun Ma, Yanqing Tie

**Affiliations:** 1 Graduate School, Hebei North University, Zhangjiakou 075000, China; leoyee2000@126.com (H.L.);; 2 Department of Clinical Laboratory, Hebei General Hospital, Shijiazhuang 050057, China; 3 National Key Laboratory of Intelligent Tracking and Forecasting for Infectious Diseases, National Institute for Viral Disease Control and Prevention, Chinese Center for Disease Control and Prevention, Beijing 102206, China; 4 Graduate School, Hebei Medical University, Shijiazhuang 050011, China

**Keywords:** HPV (human papillomavirus), LAMP (loop-mediated isothermal amplification), microfluidic chips, genotyping

## Abstract

Persistent HPV infection is a major risk factor for the subsequent development of cervical cancer. LAMP is simple and suitable for field detection in the resource-limited settings. In this study, hydroxy naphthol blue (HNB)-based visual LAMP and evagreen-based fluorescent LAMP coupled with a microfluidic chip (LAMP-chip) were established for the field detection of seven subtypes of HPV. The analytical sensitivity was 19–233 copies/reaction. The overall clinical sensitivity was 97.35% for visual LAMP and 98.23% for LAMP-chip. Both LAMP assays exhibited 100% specificity and were completed in less than 50 min. Additionally, both assays did not require complicated nucleic acid extraction and purification steps. A complete quality control monitoring system (including internal control, positive quality control and negative control) in the LAMP assays further ensured the credibility of the results. Our findings demonstrated that the proposed LAMP assays have the potential to be applied in the testing of common HPV DNA in field investigations (visual LAMP) or within communities and primary health centers (LAMP-chip).

## 1. Introduction

Cervical cancer is one of the major threats to women’s health and is the fourth most common cancer in females worldwide [1]. According to World Health Organization data, in 2022 there were 660,000 new cases of cervical cancer globally and about 350,000 women who died from this disease [2]. Human papillomavirus (HPV) is the most common sexually transmitted disease in the world and can be classified into high-risk and low-risk subtypes, with high-risk subtypes of HPV being closely related to cervical cancer, and low-risk subtypes being the causative agent of benign or low-grade cervical cell lesions [3]. And almost all cervical cancers are caused by persistent high-risk HPV infections [4]. Among the high-risk subtypes, HPV16, 18, 31, 33, 35, 45, 52, and 58 are most closely associated with cervical cancer, and in China, HPV16, 18, 33, 45, 52, and 58 are the most common high-risk subtypes. Of these, HPV16 is by far the predominant subtype, causing more than 50 percent of cervical cancers, and HPV18 and HPV45 follow behind. HPV52 and HPV58 are rare in the West, but they are common in China [5]. In addition, condyloma acuminatum is a benign tumor caused in more than 90% of cases by low-risk HPV subtypes 6 or 11 infecting the cutaneous and mucosal areas of the genitals and anus [6].

Conventionally, screening for cervical cancer is based on cytological tests [7], but it relies on morphological criteria that depend heavily on the experience of the operator and is a highly subjective test. Pap smears usually show varying sensitivities (30–87%), and in ambiguous cases, the test needs to be repeated to improve diagnoses. Moreover, the presence of cytological lesions may herald the beginning of cancer, which is not a good opportunity for early intervention. The World Health Organization has recommended HPV DNA testing as the primary screening method in its latest guidelines for screening and treating pre-cancerous lesions for the prevention of cervical cancer [8,9]. Currently, HPV DNA molecular assays such as real-time PCR (qPCR) are considered the gold standard for the identification of HPV subtypes [10]. However, qPCR is complex, time-consuming, expensive in terms of reagents and consumables and requires large-scale temperature-changing instruments and systematic training of technicians [11], which is not suitable for resource-poor clinical environments or on-site testing [12].

The loop-mediated isothermal amplification method (LAMP) is a technique for amplifying DNA under constant temperature conditions, originally designed and applied to the detection of viral and other pathogenic genes by Notomi et al. [13]. Compared to qPCR, LAMP avoids the need to use a thermal cycling device in assays [14], which is highly suitable for resource-poor clinical environments or on-site testing [15]. Previously, Masanori et al. were the first team to demonstrate the value of LAMP in HPV testing [16]. Later, Chitladda Saetiew et al. [17] and Le Luo et al. [5] developed two ways for the detection of HPV by LAMP: through the appearance of turbidity caused by the precipitation of magnesium pyrophosphate and the color change indicated by the HNB dye, respectively. Color differentiation can also be achieved by the addition of gold nanoparticles (AuNPs) [18] or pH-sensitive dyes [19], thus avoiding the reliance on dedicated machines for result reading and facilitating on-site testing. In addition, Ratchanida Kumvongpin’s team also attempted to introduce the lateral-flow dipstick (LFD) test to the LAMP analysis [20]. ZHAO X et al. [21] and MAO Z et al. [22] used LAMP combined with microfluidic chip technology for the detection of five and three high-risk HPV types, respectively. However, there are some shortcomings in the above studies: insufficient coverage of HPV subtype targets, no complete quality control monitoring systems, complex nucleic acid purification and extraction steps, or limited throughput.

Herein, we report on the development of two field-applicable LAMP assays for five high-risk subtypes (HPV16, 18, 45, 52, and 58) and two low-risk subtypes (HPV6 and 11), hydroxy naphthol blue (HNB)-based visual LAMP for field investigation and evagreen dye-based fluorescent LAMP coupled with a microfluidic chip (LAMP-chip) for utilization in communities and primary health centers.

## 2. Materials and Methods

### 2.1. Clinical Samples and Recombinant Plasmids

A total of 236 clinical genital tract secretion swab samples included positive samples—HPV6 (*n* = 29), HPV11 (*n* = 10), HPV16 (*n* = 24), HPV18 (*n* = 23), HPV45 (*n* = 9), HPV52 (*n* = 108), and HPV58 (*n* = 23)—and negative samples (*n* = 10) previously typed using commercial nucleic acid extraction and purification kits and clinically approved qPCR testing (bioPerfectus technologies, Taizhou, China) following the manufacturer’s instructions. The HPV-positive sample had a Ct value in the range of 20.32–35.76. Among them, there were 69 clinical specimens with Ct values between 20.32 and 25.50, 49 clinical specimens between 25.51 and 30.05, as well as 118 clinical specimens between 30.06 and 35.76. The absence of a typical S-type amplification curve or a CT value higher than 37 is considered a negative result for the corresponding HPV type. These clinical samples were collected from patients who visited Hebei General Hospital for HPV testing in the Department of Laboratory Medicine from 30 June 2023 to 14 April 2024. Patient types included symptomatic HPV-infected patients, asymptomatic HPV carriers, and those who visited the hospital for a physical examination. In addition, we also obtained HPV standards from the hospital to initially evaluate the LAMP assays.

The recombinant plasmids were synthesized by Tsingke Biotech Co., Ltd. (Beijing, China). The F3-B3C target gene regions of each of the seven subtypes of HPV were inserted into the plasmid vector pUC57, and the DNA sequence of the plasmid clones were confirmed by sequencing. The concentration of each plasmid was then determined by Multiskan spectrum UV spectrophotometry, and the number of copies per microliter was calculated according to the following formula: number of virus copies (copies/μL) = (6.02 × 1023) × (measured value × 10 − 9)/(DNA length × 660). After determining the copy number using the above formula, each plasmid was diluted in a 10-fold gradient to 1 × 10^0^ and measured again with Multiskan spectrum UV spectroscopy to confirm whether the copy number of each diluted tube was correct.

### 2.2. Design of Primers

The HPV16 primers are cited in our previous report [5], and the primers of the other HPV subtypes were designed as follows: The whole-genome sequences of each HPV genotype were downloaded from the NCBI website (accession numbers: HG793939.1 (HPV6); HE574705.1 (HPV11); NC_001526.4 (HPV16); GQ180792.1 (HPV18); LR862061.1 (HPV45); LR861906.1 (HPV52); LR862076.1 (HPV58)). The HPV gene sequences were compared with each other using Vector NTI Advance 11.5.1, and the gene segments with fewer variations were selected for a BLAST (Basic Local Alignment Search Tool) comparison, and highly specific HPV sequence fragments were chosen. PrimerExplorer V5 (http://primerexplorer.jp/lampv5e/index.html (accessed on 22 May 2023)) and LAMP DESIGNER 1.16 were used to design the primers, and AmplifX 1.7.0 and Oligo 7.56 were used to initially evaluate the designed primers. A degenerative base was introduced in the primers, if necessary, to improve their sensitivity. An internal control primer targeting β-actin (ACTB) was cited to monitor whether the nucleic acid extraction was in place or whether the sample was qualified [23]. All the primers were synthesized with the assistance of Sangon Biotech (Shanghai, China), and the sequence of each primer is shown in Table 1.

### 2.3. Simple and Efficient Sample Lysis

We used DNA-releasing reagents (Amp-future, Changzhou, China) for the rapid nucleic acid extraction of all clinical samples. Firstly, the HPV clinical sample was shaken and rested, and 20 μL of liquid sample was added into a centrifuge tube containing 80 μL of DNA-releasing reagents (sample:reagent = 1:4) and then added into the reaction system for detection after resting for 3 min at room temperature or stored in a −80 °C freezer.

### 2.4. Optimization of LAMP Reaction Conditions

To optimize LAMP reaction conditions, we performed evagreen-based LAMP reactions on a real-time PCR instrument (ROCGENE, Beijing, China) that detects fluorescent signals. We tested the amplification efficiency of LAMP assays at different concentrations of ions and additives (MgSO_4_, dNTP) as well as at different temperatures (Supplementary Figure S1). Considering the amplification efficiency and cost-effectiveness, finally, the volume of evagreen-based LAMP reaction solution designed in this experiment was 25 μL, containing final concentrations of 1X Buffer, 0.5 M Betaine (Sigma-Aldrich Co., St. Louis, MO, USA), 1X Evagreen (Biotium, SF Bay Area, CA, USA), 3 mM MgSO_4_, 1.2 mM dNTPs, 0.2 μM F3/B3 primer, 1.6 μM FIP/BIP primer, 0.8 μM LF/LB primer, and 0.32 U/μL Bst 3.0 DNA Polymerase (NEW ENGLAND Biolab, Ipswich, MA, USA). The HNB-based LAMP reaction solution for this experiment was 25 μL, containing a final concentration of 1X Buffer, 0.5 M Betaine, 0.12 mM HNB (Tianjingsha Gene Technology Co., Beijing, China), 6 mM MgSO_4_, 1 mM dNTPs, 0.2 μM F3/B3 primer, 1.6 μM FIP/BIP primer, 0.8 μM LF/LB primer, and 0.32 U/μL Bst 3.0 DNA Polymerase. All the LAMP reactions were incubated at 63 °C for 50 min.

### 2.5. Specificity and Sensitivity of LAMP Assays for HPV Detection

We validated the primer specificity of both the HNB-based and evagreen-based LAMP assays for the seven HPV subtypes. In the 8-strip tubes used, the primers added to the eight wells were the same for the specificity measurement, and the nucleic acids added were the DNA of the seven HPV subtypes samples extracted using DNA-releasing reagents and water used as a negative control, respectively. The validation test was repeated three times.

We also validated the primer sensitivity of both HNB-based and evagreen-based LAMP assays for the seven HPV subtypes. In the 8-strip tubes used, the primers added to the eight wells were the same primers for the sensitivity measurement, and the nucleic acids added were the DNA of the synthetic plasmids of the corresponding subtype genes in seven 10-fold dilutions of concentrations 10^0^–10^6^ (copies/μL) and water as a negative control, respectively. The validation test was repeated eight times and probit analysis was performed on the results.

### 2.6. LAMP Detection Coupled with a Microfluidic Chip (LAMP-Chip)

**Figure 1:**
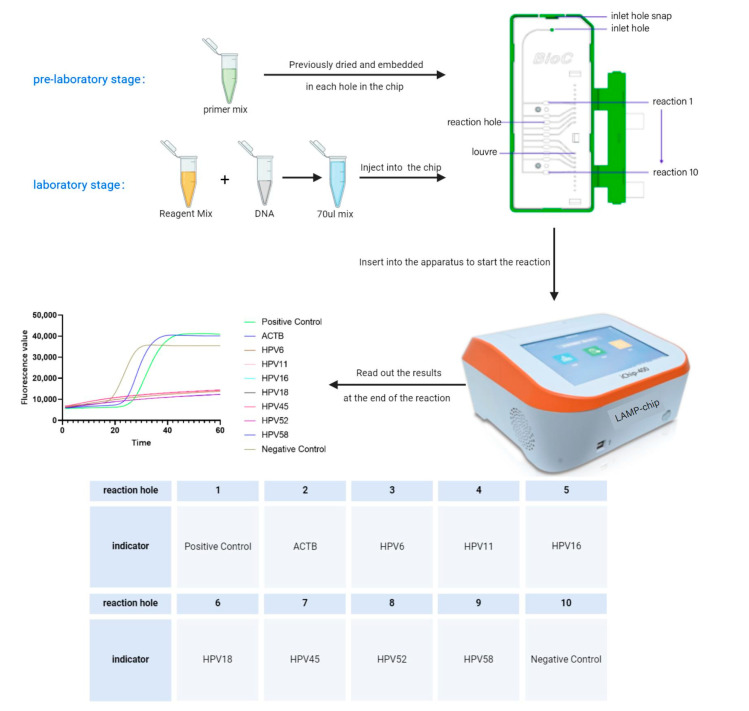
The procedure of LAMP on microchip and the structure of the microfluidic chip and the corresponding instruments (Baicare Company, Beijing, China) as well as the detection indicators corresponding to the 10 reaction wells.

We collaborated with Baicare Company (Beijing, China) for the fabrication of microfluidic microarrays. Firstly, we formulated 6 primers for each HPV subtype into a primer mix (primer final concentration of FIP, BIP:1.6 μM; F3, B3:0.2 μM; LB, LF:0.8 μM), and then dried and embedded them into the reaction wells of the microchip. The detection indicators corresponding to the 10 reaction wells are shown in Figure 1. Valid assay results were only obtained when the positive control and ACTB internal control in reaction wells 1 and 2 were amplified normally to ensure that the reagents and kits worked properly and that the nucleic acid extraction was satisfactory.

The LAMP amplification reagents were proportionally mixed with nucleic acids in 70 μL and injected into the chip, and the reaction was carried out by running the established procedure (63 °C, 50 min) on the instrument iChip^®^ 400 (Baicare Company, Beijing, China). The operation procedure of LAMP-chip, as well as the structure of the microfluidic chip and the corresponding instrument are shown in Figure 1. This instrument could detect four samples (four chips) simultaneously at any one time.

### 2.7. Detection of Clinical Samples and Statistical Analysis

Totally, 236 clinical genital tract secretion swab samples typed previously by the qPCR-HPV methods were examined by the visual LAMP based on HNB dye and microfluidic chip LAMP based on evagreen dye.

All statistical analyses were performed with IBM SPSS Statistics 27 (IBM Corporation, Armonk, NY, USA). We determined the 95% probability detection limit of the LAMP assays using probit analysis, and Kappa values (κ) were used to measure the agreement between the LAMP assays and qPCR results. A *p*-value of <0.05 indicated a statistically significant difference.

## 3. Results

### 3.1. Selection of Primers and HPV Standard Assessment

We designed 1–3 sets of primers for each subtype of HPV and ultimately selected the set of primers with high specificity, no cross-reactivity between subtypes, and high amplification efficiency (as shown in Table 1). Prior to evaluation using plasmids and clinical specimens, we performed an initial evaluation using HPV standards, which showed 100% specificity and high sensitivity.

### 3.2. Specificity of Two LAMP Assays for HPV Detection

**Figure 2:**
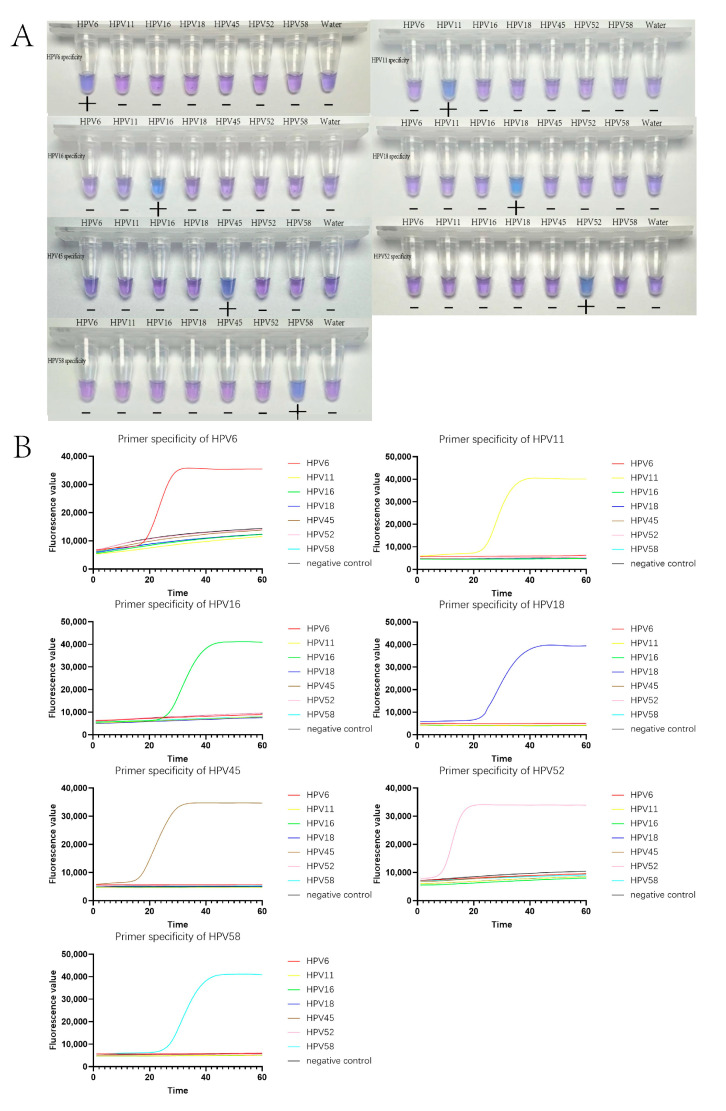
(**A**) The specificity of HNB-LAMP for HPV detection. The HNB dye was blue when the reactions were positive and purple when the reactions were negative. (**B**) The specificity of HPV detection using Evagreen-LAMP. The amplification curves showed a smooth “S” shape with a fluorescence value of over 30,000 determined as positives. If there was no typical S-amplification curve or the CT value was higher than 37, the result was determined to be negative.

The specificity of the primers for each subtype of HPV was assessed using HNB-LAMP and evagreen-LAMP. In the eight-strip tubes used, eight wells were added with different primers corresponding to seven HPV subtypes and water as a negative control. In this part, each subtype was validated with three clinical specimens (twenty-one clinical specimens in total), and the validation test was repeated three times. The results of the test are shown in Figure 2. These results indicated that each LAMP primer could definitively detect specific subtypes of HPV with high specificity and did not cross-react with other subtypes of HPV DNA.

### 3.3. Sensitivity of Two LAMP Assays for HPV Detection

**Figure 3:**
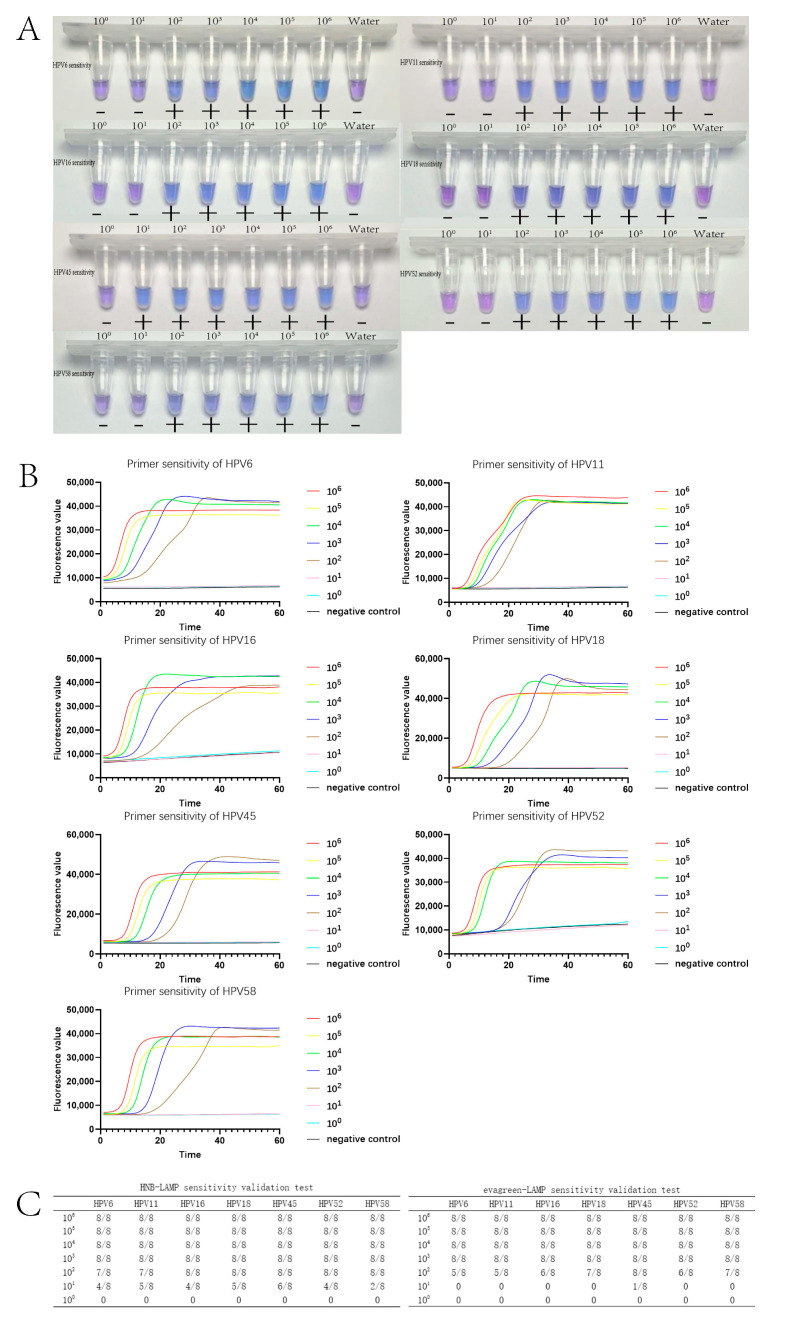
(**A**) Sensitivity of HNB-LAMP for HPV detection. The HNB dye was blue when the reactions were positive and was purple when the reactions were negative. (**B**) Sensitivity of evagreen-LAMP for HPV detection. The amplification curves showed a smooth “S” shape with a fluorescence value of over 30,000 determined as positives. If there was no typical S-amplification curve or the CT value was higher than 37, it was determined to be negative. (**C**) The results of the HNB-LAMP and evagreen-LAMP sensitivity validation tests. The vertical columns indicate the concentrations of the plasmids, and the horizontal rows correspond to each type of HPV.

Primer sensitivity was validated for seven subtypes using HNB-LAMP and evagreen-LAMP. In the eight-strip tubes used, the primers added to the eight wells were the same for the sensitivity measurement. The nucleic acids added were the synthetic plasmid DNA corresponding to each HPV subtype in seven 10-fold dilutions of concentrations ranging from 10^0^ to 10^6^ (copies/μL) and water as a negative control, respectively. In this section, each subtype sensitivity validation test was repeated eight times. The experimental results are shown in Figure 3. We used the data from the results of eight sensitivity experiments for each HPV subtype (Figure 3C) for probit analysis, and the sensitivity of the HNB-LAMP assay for each HPV subtype was 19–146 copies/reaction while the sensitivity of the evagreen-LAMP assay for each HPV subtype was 74–233 copies/reaction.

### 3.4. Clinical Evaluation and Comparison between the LAMP Assays and qPCR

As shown in Table 2, the sensitivity of HNB-LAMP for the HPV 6, 11, 16, 18, 45, and 52 and 58 clinical samples was 100%, 100%, 95.83%, 95.65%, 100%, 96.30%, and 100%, respectively. Compared with qPCR, HNB-LAMP had 97.35% sensitivity and 100% specificity. We obtained a positive predictive value (PPV) of 100%, a negative predictive value (NPV) of 62.5%, an overall coincidence rate of 97.46%, and a kappa value of 0.757 (*p* < 0.05) (Table 3).

Based on the good sensitivity and specificity performance of evagreen-based LAMP, we then applied the amplification system of evagreen-based LAMP in proportional ratios to the microfluidic chip with a portable instrument. As shown in Table 2, the sensitivity of LAMP-chip for the HPV 6, 11, 16, 18, 45, 52, and 58 clinical samples was 96.55%, 100%, 95.83%, 100%, 100%, 98.15%, and 100%, respectively. Compared with qPCR, LAMP-chip had 98.23% sensitivity and 100% specificity. We obtained a PPV of 100%, an NPV of 71.43%, an overall coincidence rate of 98.31%, and a kappa value of 0.825 (*p* < 0.05) (Table 4).

As mentioned above, the percentage sensitivity of HNB-LAMP and LAMP-chip exceeded 96.5%, and the specificity was 100% compared to qPCR, which implies that both LAMP assays are able to detect HPV well and minimize leakage and misdiagnosis. The PPVs were all 100% indicating that there were no false-positive results in this experiment. The NPVs of 62.5% and 71.43% were not high, probably because the number (only 10) of negative specimens was too small, while the number (226) of positive samples was much larger than the number of negative samples. The overall concordance rate exceeded 97.4% with Kappa values of 0.757 (HNB-LAMP) and 0.825 (LAMP-chip), indicating high concordance between the results of the LAMP assays and qPCR for the detection of HPV DNA, further suggesting that LAMP assays in this study are able to detect HPV DNA well.

## 4. Discussion

The significance of HPV is not only related to cervical cancer and condyloma acuminatum, but also to lung cancer [24], head and neck neoplasms [25], skin cancer [26], and periodontitis [27]. In China, the age of HPV-infected patients shows a “bimodal distribution” during puberty and perimenopause, and there is a trend towards younger age groups [28]. Therefore, it is important to enhance the regular screening and increase HPV screening rates.

In this study, two field-applicable approaches based on HNB-LAMP and LAMP-chip were established to detect five high-risk subtypes of HPV (HPV subtypes 16, 18, 45, 52, and 58) and two low-risk subtypes (HPV subtypes 6 and 11). The specificity and sensitivity of the LAMP assays were assessed using both the recombinant plasmids of HPV DNA and 236 clinical genital tract secretion samples typed previously by the qPCR-HPV method. The results of the LAMP assays were also compared and statistically analyzed with the qPCR results.

Both the assays achieved 100% specificity and over 97% sensitivity compared to qPCR. The HNB-LAMP reaction takes only 40–50 min, and the results can be determined by the naked eye only at the end of the experiment while LAMP-chip takes only 20–50 min, and the results are determined by fluorescence curves in real time. In comparison with the reported LAMP assays, our study shows a number of advantages: the whole process does not require a complex step of extracting nucleic acid nor the need to open the tube after amplification so as to avoid contamination. The simplified DNA extraction process shortens the operation time from 15 min at 100 °C to 5 min at room temperature, making the assays more convenient and easier to work with. In addition, a full range of internal control and positive quality control as well as negative control further ensure the credibility of the experiment.

HNB-LAMP is a feasible on-site testing method, which can visually confirm the LAMP results based on the color change with low requirements for experimental conditions and instrumentation. However, the need for LAMP to react in tubes with sample volumes of tens or even hundreds of microliters not only limits further implementation of the technology but also presents difficulties in detection in micro samples [29]. Microfluidic chips can process just a few microliters of solution within a microtubular channel and are capable of simultaneous reaction control, detection, and evaluation of results within a single chip. The use of microfluidic chips therefore greatly improves the detection capability of LAMP, providing further cost savings and enabling high-throughput, low-cost simultaneous detection of multiple targets [30].

Nowadays, patients who register for a test at a hospital usually have to wait until the next day or 3–4 h for the results and then go back for a follow-up appointment or even receive the test again, which is a cumbersome process that significantly reduces their willingness to seek healthcare, especially in areas with limited access to healthcare. The seven-subtype-specific LAMP in this study enables patients to obtain results in a single test, and these subtypes can be detected in most cases in China. POCT (point-of-care technology) is a technology that is performed instantly at the patient’s place of visit (such as a healthcare facility, clinic, home, etc.), and the results are usually available within a short period of time [31]. Combining LAMP with chip for HPV detection, such as the microfluidic microarray device described in this paper, with its rapid response rate, simple procedure, and easy portability will significantly increase the willingness of patients to be tested and thus improve the effectiveness of cervical cancer prevention, especially in remote and underdeveloped areas. Therefore, the proposed LAMP-chip is more suitable for community, primary health center, or in-hospital testing.

There are some noteworthy aspects of this study. Firstly, visual HNB-LAMP is an end-point method; the results are relatively subjective due to inherent differences in visual perception from person to person [32]. In some cases, the testing of the clinical specimen may result in an insignificant color change, or it may be susceptible to the interference of the color of the clinical sample itself. Secondly, the determination of fluorescent LAMP results is susceptible to interference from elevated curves caused by primer dimerization or contamination [33], but its interference can be eliminated by the fluorescence value and curve shape. Thirdly, the Ct values of the LAMP assays were negative, but the qPCR-positive samples (*n* = 10) were concentrated in the range of 20.35–25.50; this discrepancy is more likely due to the nucleic acid degradation of the samples themselves as samples with a Ct value of 20.32 could also be detected positively by LAMP assays. Lastly, in Table 1, the sequences of HPV6-B3 and HPV11 are partially identical, as well as the sequences of HPV11-F3 and HPV6, but the LAMP amplification reaction is based on six primers. If only one primer binds to the target DNA, the amplification reaction cannot proceed. We conducted 236 clinical sample evaluation experiments and passed the specificity test, the results indicated no cross-reactivity between HPV subtypes, and there were no false-positive reactions observed.

However, there are shortcomings in this study. The first is that more clinical samples are needed for a more comprehensive assessment. The second is that, as mentioned above, HPV 16, 18, 33, 45, 52, and 58 are the most common high-risk subtypes in China. Considering the number of clinical HPV-positive samples and the throughput limitation of eight-strip tubes, we finally chose seven subtypes for testing except HPV33, as these seven subtypes can be detected in most of the cases in China. The third is that the *Alphapapillomavirus* 7 strain African L1 protein gene has more than 99% similarity to the HPV18 L1 protein gene, and *Alphapapillomavirus* 12 strain RhPV KM1 L1 protein (L1) gene has more than 98% similarity to the HPV52 L1 protein gene. Therefore, when using HNB-LAMP or LAMP-chip to test clinical samples that show positive results for HPV18 or HPV52, the possibility of *Alphapapillomavirus* 7 or *Alphapapillomavirus* 12 infection should be additionally considered. The last is that more controls of HPV’s closely and distantly related viruses are needed to further validate the specificity of the proposed LAMP assays.

## 5. Conclusions

We established two rapid and accurate field-applicable LAMP assays for seven common HPV subtypes. No complicated nucleic acid extraction and purification steps are required, and the results can be observed with the naked eye (HNB-LAMP) or a small portable microfluidic instrument (LAMP-chip) with a complete quality control system in less than 50 min. The proposed LAMP assays have the potential to be applied in the testing of common HPV DNA in field investigations (HNB-LAMP) or within communities and primary health centers (LAMP-chip), which offers a valuable tool for early screenings of HPV, prevention of cervical cancer, and other aspects of women’s health.

## Figures and Tables

**Table 1:** Primer sequences and information.

Primer	Amplification Region	Amplicon Length	Sequences(5′→3′) ^1^
HPV6-F3	E6	204	GGATATGCAACAACWGTTGAAG
HPV6-B3 ^2^			TTAGGGTAACAWGTCTTCCAT
HPV6-FIP			TCTACTTCACACAGCGGTTTGTCAAGACATYTTAGACGTGCTAA
HPV6-BIP			ACTAACCAAGGCACGGTTYATCATGTTGTCCAGCAGTGT
HPV6-LF			GACACAGGTAGCACCGAA
HPV6-LB			ATTGTACGTGGAAGGGTCG
HPV11-F3 ^2^	E6	198	GTAAAGATGCCTCCACGT
HPV11-B3			CTAAGCAACAGGCACACG
HPV11-FIP			CCTGCAAAACACGCACTGAAGACCAGTTGTGCAAGACG
HPV11-BIP			ACTGACCACCGCAGAGATATAAGGGAAAGTTGTCTCGC
HPV11-LF			GCAGAGTGTGCAAAGAAAG
HPV11-LB			GCATATGCCTATAAGAACC
HPV16-F3	E7	189	AGACAACTGATCTCTACTGTT
HPV16-B3			CTTCCAAAGTACGAATGTCTAC
HPV16-FIP			TTCTGCTTGTCCAGCTGGACGCAATTAAATGACAGCTCAGAG
HPV16-BIP			CCGGACAGAGCCCATTACAATGTGTGTGCTTTGTACGCA
HPV16-LF			CATCTATTTCATCCTCCTC
HPV16-LB			TGCAAGTGTGACTCTACGCT
HPV18-F3	L1 ^3^	207	CGCGTCCTTTATCACAGG
HPV18-B3			TGGAATCCCCATAAGGATC
HPV18-FIP			GGCACCATATCCAGTATCTACCATAATTGCCCCCCTTTAGAACT
HPV18-BIP			TGCAAGATACTAAATGTGAGGTACCGCAGACATTTGTAAATAATCAGGAT
HPV18-LF			TCACCATCTTCCAAAACTG
HPV18-LB			ATTGGATATTTGTCAGTCT
HPV45-F3	L1	220	ACTAAGTTTAAGCASTATAGTAGAC
HPV45-B3			CCTTTTGACAGGTAACAGC
HPV45-FIP			ATGACATAACCTCTGCAGTTAAAGTTGTGGAGGAATATGATTTACAGTT
HPV45-BIP			AATTGGAATTTTGGTGTMCCTCCACTGATTGCACAAAACGATA
HPV45-LF			AGTGCACAACTGAAAA
HPV45-LB			ACCACCTACTACAAGTTTRGTGGA
HPV52-F3	L1 ^3^	193	GGCAATACTGCCACTGTAC
HPV52-B3			ATAAAGTCATGTTAGTGCTACG
HPV52-FIP			ACGTTGTAACCAGTACGGTTTATTAAAGCAGTGCTTTTTTTCCTAC
HPV52-BIP			CAGGGCCACAATAATGGCATTGTGTGAGTGGTATCCACAACTGTGA
HPV52-LF			GGGATTCTGAGGTTACCATAGAACC
HPV52-LB			GTTGGGGCAATCAGTTGTTTG
HPV58-F3	E7	238	ACATCCTGAACCAAYTGACC
HPV58-B3			GCTRGGGCACACAATRGTAC
HPV58-FIP			CTGTGGCCGGTTGTGCTTGTTTTTTGTGACAGCTCAGACGAGG
HPV58-BIP			TACACTTGTRRCRCCACGGTTTTTTCCCATAAGCAGCTGCTGTAG
HPV58-LF			CCATCTGGCCCGTCCAA
HPV58-LB			GTGTATCAACAGTACARCAACYGAM
ACTB-F3		203	GCTCAGGGCTTCTTGTCC
ACTB-B3			TCGGGAGCCACACGCA
ACTB-FIP			TTGCTCTGGGCCTCGTCGCTTTTTTTCCTTCCCAGGGCGT
ACTB-BIP			AGAGGCATCCTCACCCTGAAGTTTTTGTGGTGCCAGATTTTCTCCA
ACTB-LF			TGACCCATGCCCACCATC
ACTB-LB			CCATCGAGCACGGCATC

^1^ In these primers, degenerate bases are used, where “W” represents “A, T” bases; “Y” represents “C, T” bases; “M” represents “A, C” bases; and “R” represents “A, G” bases. ^2^ The sequence of HPV6-B3 is partially identical to that of HPV11, and the sequence of HPV11-F3 is also partially identical to that of HPV6. ^3^ The *Alphapapillomavirus* 7 strain African L1 protein gene has more than 99% similarity to the HPV18 L1 protein gene, and the *Alphapapillomavirus* 12 strain RhPV KM1 L1 protein (L1) gene has more than 98% similarity to the HPV52 L1 protein gene.

**Table 2:** Experimental results of HNB-LAMP and LAMP-chip for detecting HPV in clinical samples and comparison with qPCR.

HPV Subtype Samples	Results of HNB-LAMP/Results of qPCR	Results of LAMP-Chip/Results of qPCR	Total
HPV6	29/29	28/29	236
HPV11	10/10	10/10	
HPV16	23/24	23/24	
HPV18	22/23	23/23	
HPV45	9/9	9/9	
HPV52	104/108	106/108	
HPV58	23/23	23/23	
Negative sample	10	10	

**Table 3:** Sample evaluation comparison between HNB-LAMP and qPCR. PPV—positive predictive value; NPV—negative predictive value.

HNB-LAMP	qPCR		Total	Sensitivity	Specificity	PPV	NPV	Kappa
	+	−						
+	220	0	220	97.35%	100%	100%	62.5%	0.757
−	6	10	16					
Total	226	10	236					

**Table 4:** Sample evaluation comparison between LAMP-chip and qPCR. PPV—positive predictive value; NPV—negative predictive value.

LAMP-Chip	qPCR		Total	Sensitivity	Specificity	PPV	NPV	Kappa
	+	−						
+	222	0	222	98.23%	100%	100%	71.43%	0.825
−	4	10	14					
Total	226	10	236					

## Data Availability

The datasets used and/or analyzed in the current study can be provided on reasonable request. All requests should be made to the corresponding authors.

## Supplementary Materials

The following supporting information can be downloaded at: https://www.mdpi.com/article/10.3390/tropicalmed9100240/s1, Figure S1. Optimization of the LAMP reaction conditions. A: Amplification curves of evagreen-based LAMP with dNTPs concentration gradient (0.6 mM–1.6 mM). B: Amplification curves of evagreen-based LAMP with temperature gradient (61 °C–69 °C). C: Amplification curves of evagreen-based LAMP with MgSO_4_ concentration gradient (0 mM–5 mM).
