# Supplementary material for: Field-Applicable Loop-Mediated Isothermal Amplification for the Detection of Seven Common Human Papillomavirus Subtypes
Source: Trop Med Infect Dis. 2024 Oct 12;9(10):240. doi: 10.3390/tropicalmed9100240 (PMC11511265; doi:10.3390/tropicalmed9100240)
Supplement: Supplementary file 1 [file tropicalmed-09-00240-s001.zip › tropicalmed-3179280-supplementary.pdf]

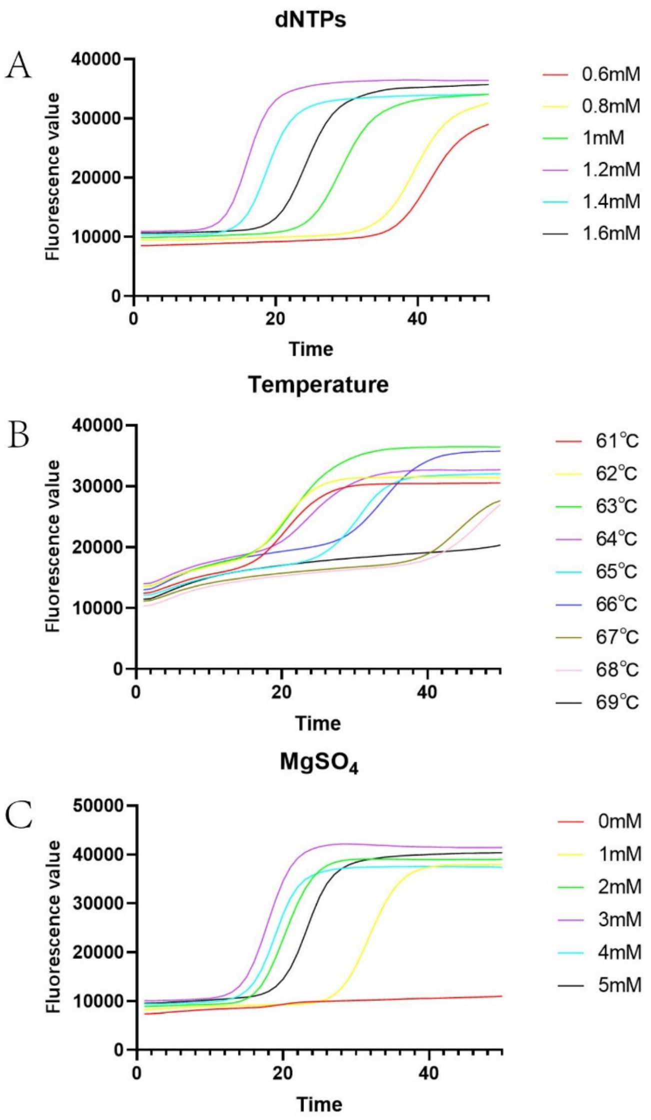

Figure S1. Optimization of the LAMP reaction conditions. A: Amplification curves of evagreen-based LAMP with dNTPs concentration gradient (0.6 mM-1.6 mM). B: Amplification curves of evagreen-based LAMP with temperature gradient (61℃-69℃). C: Amplification curves of evagreen-based LAMP with $MgSO_4$ concentration gradient (0mM-5mM).
